# Preparation and Molecular Structural Characterization of Fulvic Acid Extracted from Different Types of Peat

**DOI:** 10.3390/molecules28196780

**Published:** 2023-09-23

**Authors:** Di Wu, Yanan Lu, Litong Ma, Jianguo Cheng, Xiaoxia Wang

**Affiliations:** 1 School of Chemistry and Chemical Engineering, Inner Mongolia University of Science and Technology, Baotou 014010, China; wudi990821@126.com (D.W.); lyn60710@163.com (Y.L.); cjg143@126.com (J.C.); wxx572369@163.com (X.W.); 2 Inner Mongolia Engineering Research Center of Comprehensive Utilization of Bio-Coal Chemical Industry, Baotou 014010, China; 3 Laboratory of Low Rank Coal Carbon Neutralization, Inner Mongolia University of Science and Technology, Baotou 014010, China

**Keywords:** peat, fulvic acid, spectral analysis, molecular structure

## Abstract

Humic acid is a type of polymeric, organic weak acid mixture with a core aromatic structure and main-component oxygen-containing functional group. Fulvic acid is a type of humic substance that can be dissolved in acid, alkali, or water. This study discusses the influence of different peptides on the molecular structure of fulvic acid, which was extracted from herbaceous, woody, and mossy peats using alkaline dissolution and acid precipitation methods. Analyses using infrared, UV-Vis, ^13^C-NMR, and X-ray photoelectron spectroscopies, as well as X-ray diffraction (XRD), were conducted to compare the effects of different peat types on the content and molecular structure of fulvic acid. The woody peat fulvic acid content was the highest among all peat fulvic acids (0.38%). However, the yield of fulvic acid from herbaceous peat was the highest (2.53%). Herbaceous peat fulvic acid contains significant quantities of carbonyl, amino, methylene, carboxyl, and phenolic hydroxyl groups and ether bonds. Woody peat fulvic acid contains carbonyl and methoxy groups, benzenes, aromatic carbons, aromatic ethers, and phenols. The degree of aromatization of woody peat fulvic acid was the highest. Mossy peat fulvic acid contains high levels of hydroxy, methyl, methylene, and phenol groups and aromatic ethers. The structural differences in fulvic acids in the different types of peat were primarily manifested in the content of functional groups, with little influence from the types of functional groups. XRD analysis of the different peats revealed that their structures all comprised benzene rings. However, mossy peat contained more C=O and –COOH groups, whereas herbaceous peat contained more C–O groups.

## 1. Introduction

Peat, a body of organic matter, is formed by the long-term accumulation of swamp plant residues under conditions of excessive moisture, insufficient air and decomposition, and low temperatures. Carboniferous plants form peat via biodegradation under anoxic conditions, and peat forms lignite via diagenesis [1]. When the temperature and pressure gradually increase, the peat changes into bituminous coal through metamorphism until it becomes anthracite. Different species of peat-forming plants produce different types of peat, including herbaceous, woody, and mossy peats [2,3,4]. The primary components of peat are cellulose, hemicellulose, lignin, and humic substances [5].

In 1786, the German chemist Achard first extracted humic acid from peat using an alkali [6]. He found that the humus he extracted not only contained humic acid, but also fulvic acid. This macromolecular substance contains a benzene ring as the basic unit, which is connected to an oxygen bridge(–O–), methylene group (–CH_2_–), –CH_2_–CH_2_–, –NH–, and –S–. Numerous active functional groups, such as carboxyl, hydroxyl, and methoxy groups, are found on the benzene ring and side chain [7,8,9,10] because of their small molecular weights and high solubilities, thereby endowing fulvic acid with greater physiological activities than that of ordinary humic acid. Kattamachi Gnananath et al. [11]. designed a project to enhance the proposition of adding humic acid as a functional excipient. Research has found that humic acid enhances the solubility and/or bioavailability of different BCS Class II drugs. It is a plant growth regulator, of which the plant growth can be promoted, and plays an important role in combating drought, enhancing plant stress resistance, increasing yield, and improving quality [12]. Peat fulvic acid has a small molecular weight, simple structure, and rich active groups, which renders its activity and antiflocculation abilities far superior to those of lignite and weathered-coal fulvic acid. Therefore, peat fulvic acid is primarily used in high value-added, water-soluble fertilizers and in the medical field. Fulvic acid, an active humic substance, has a relatively low molecular weight and contains a high amount of oxygen-rich and carbon-poor functional groups [13,14]. Many studies have shown that FA has many plant physiological activities. FA significantly alleviated the toxic symptoms of Cd on lettuce seedlings [15], and protected soybean and barley against salt stress [16,17]. 

Ran et al. [18] studied the effects of fulvic and humic acids extracted from composted straw and cow manure, peat moss, and lignite on Hg-methylation and bioenrichment in paddy soil. They found that adding fulvic and humic acids significantly increased the abundances of Hg-methylated micro-organisms and low-molecular-weight organic matter (such as cysteine) in the paddy soil. The addition of fulvic acid changed the aromatics, molecular size, and chromium concentrations of dissolved organic matter in the soil and had a heterogeneous effect on the migration and transformation of Hg. Therefore, fulvic acid improves the fluidity and methylation of Hg in soils. Different sources of fulvic acid have different effects on Hg transport in rice. Niews et al. [19] reported that ultrasound-assisted alkaline extraction of humic and fulvic acids can improve their yields when extracted from peat compared to that of traditional methods, with the highest yields attaining 56.70% and 40.55%, respectively. Fourier-transform infrared (FTIR) spectroscopy revealed the vibrations of functional groups that were characteristic of the fulvic acid fractions. The signal from the aliphatic structure was dominant. Only C=O stretching was observed in the hydrophobic (HPO) fulvic acid samples, which were extracted during low-intensity ultrasound-assisted alkaline extraction. The lack of a peak at approximately 1700 cm^−1^ for other samples may be due to the C=O vibration caused by the blockage of aliphatic chains, which dominate the HPO samples analyzed. Huculak-Mączka et al. [20] used simplified conventional and ultrasound-assisted methods to evaluate the fulvic acid components extracted from peat and lignite. They determined that the fulvic acid obtained from peat had a more aliphatic structure, higher aromatic carbon ratio, and lower carbonyl carbon ratio than that obtained from lignite. Among the fractions obtained via conventional extraction, the carbonyl group showed the largest difference. Compared to the traditional method, the ultrasound-assisted extraction (UAE) method lowered the ratio of carbon in carbonyl groups by 8.4 pp and increased the ratios of aromatic and aliphatic carbon by 3.5 and 4.9 pp, respectively, for fulvic acid obtained from lignite. For fulvic acid obtained from peat, the UAE method had less impact in terms of structural changes. Thermal analysis showed that the products were thermally stable up to 100 °C, and the simplified extraction resulted in the creation of mineral–organic structures that decomposed at unusually high temperatures. Simplifying the extraction process by excluding inorganic purification and protonation of the obtained fulvic acid fractions considerably affected the product quality and limited its possible application.

Ma et al. [21] found that the fulvic acid content in herbaceous peat pretreated with dilute sulfuric acid decreased from 5.33% to 4.19%; however, the content of –SO_3_H in fulvic acid increased. Laurynas et al. [22] identified numerous acetyl hydroxamic, lactic, and glycolic acid derivatives in peat extracts. Comparing the products after active impregnation and ultrasonic treatment, the former was found to produce organic matter more efficiently, resulting in maximum yields of 1% for fulvic acid and 15.3% for humic acid and humin. Lu et al. [23] used alkali dissolution and acid precipitation to collect humic and fulvic acids from herbaceous peat; however, different types of acid precipitation were used for extraction. These different acid treatments resulted in different structures of fulvic acid. For example, fulvic acid treated with sulfuric acid contained –SO_3_H, whereas fulvic acid treated with nitric acid contained –NO_2_. Perminova et al. [24,25] used ultrafiltration high-performance liquid chromatography to determine the partition coefficients of the binding affinity between atrazine and 16 different humic substances. Sources include humic acid, fulvic acid, as well as humic acid and fulvic acid combination components from soil, peat, and coal humic acids. Through research, it was found that the characteristic of each humic material lies in its elemental composition, and the characteristic of each humic material lies in its elemental composition, molecular weight, and the composition of the main structural fragments determined by ^13^C solution NMR. The size of K (OC) value ranges from 87 to 575 L/kg C, indicating a low binding affinity of humus for deoxyribonucleic acid. However, at the level of extraction and molecular structure analysis of fulvic acid from peat, few people have summarized the theory.

The extraction of fulvic acid from peat is necessary for peat utilization. Current research focuses on the extraction of fulvic acid from herbaceous peat. Peat-forming plants, such as herbaceous, woody, and mossy plants, determine the material composition and physicochemical properties of peat, thereby affecting the full utilization of the resources [26]. However, systematic and in-depth research on the extraction of fulvic acid from herbaceous, woody, and mossy peats is limited. Scholars from various countries are currently striving to explore the extraction of humic acid and other substances from peat, and scientific theories have been established. However, at the level of extraction and molecular structure analysis of fulvic acid from peat, few people have summarized the theory. This article provides a large amount of structural analysis of peat fulvic acid, laying the foundation for the theoretical formation of fulvic acid.

Herein, an experimental study was conducted to extract fulvic acid from herbaceous, woody, and mossy peats. The effects of the preparation of fulvic acid from these peats on its yield, content, and molecular structure were analyzed. In addition, the effects of different types of peat on the content, structure, and yield of fulvic acid were compared using infrared, UV–Vis, and nuclear magnetic resonance (NMR) spectroscopies, as well as X-ray photoelectron spectroscopy (XPS) and X-ray diffraction (XRD) to further study the molecular structure differences of different peat fulvic acids.

## 2. Results and Discussion

### 2.1. Yield and Content of Fulvic Acid from Different Types of Peat

**Figure 1:**
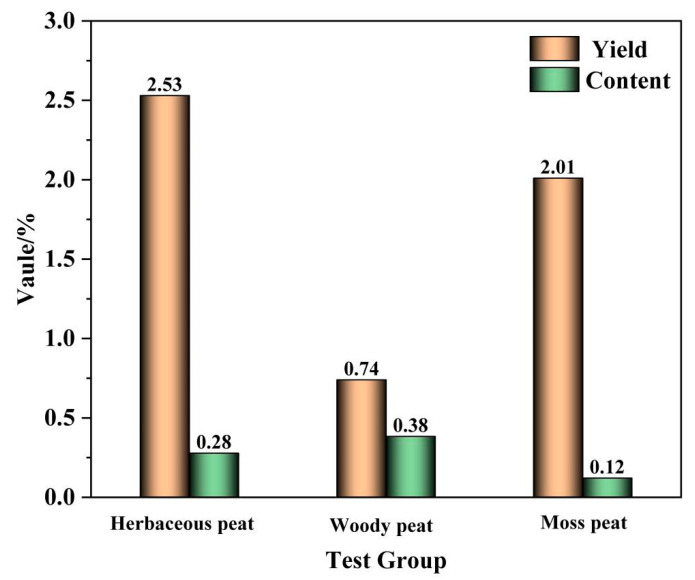
Yields and contents of fulvic acid from herbaceous, woody, and mossy peats.

Figure 1 shows the yields and contents of fulvic acid in herbaceous, woody, and mossy peats. The yield of fulvic acid extracted from herbaceous peat was the highest (2.53%), and the yield of fulvic acid extracted from woody peat was the lowest (0.74%). These results indicate that different types of peat have a significant effect on the fulvic acid yield. The fulvic acid contents in herbaceous, woody, and mossy peats were also significantly different. Woody peat had the highest fulvic acid content (0.38%) and mossy peat had the lowest (0.12%). In summary, herbaceous peat had the highest yield of extracted fulvic acid and the second highest fulvic acid content. Therefore, compared to woody and mossy peats, herbaceous peat is the best raw material for fulvic acid.

### 2.2. UV–Vis Spectra of Fulvic Acid Extracted from Different Types of Peat

**Figure 2:**
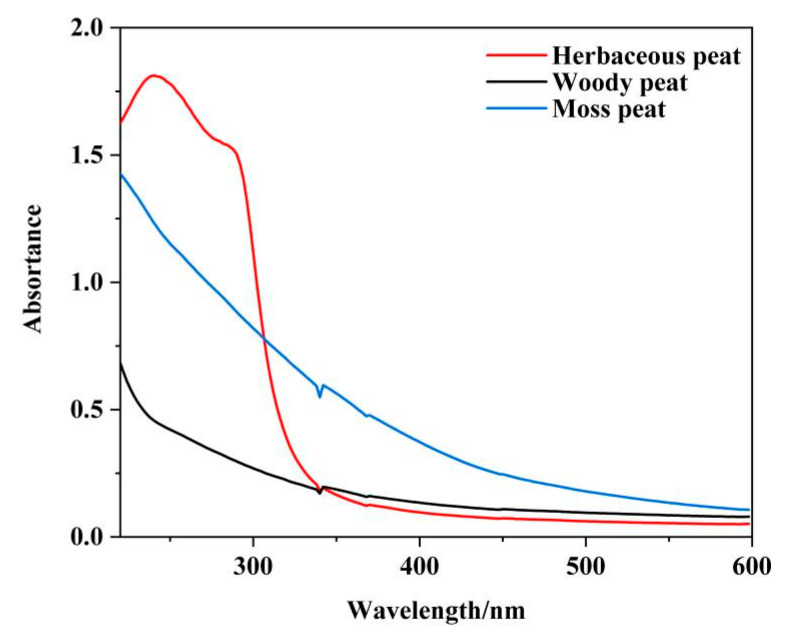
UV–Vis spectra of fulvic acid from herbaceous, woody, and mossy peats.

The UV-Vis spectra, shown in Figure 2, reveal the effects of different peat types on fulvic acid extraction. Fulvic acid contained conjugated double bonds and oxygen-containing functional groups and absorbed in the UV–Vis range. The absorbance values of fulvic acid in woody and mossy peats showed similar trends: decreasing with increasing wavelength. The peak position of herbaceous peat fulvic acid moved to red, showing a trend of first increasing and then decreasing. This may be related to the different structures and contents of conjugated bonds, simple aromatic rings, or oxygen-containing functional groups in fulvic acids from different sources.

Table 1 shows that carboniferous plant species have obvious influence on the molecular structure of peat fulvic acid. ΔLogK is the hue coefficient, recording the absorbance at 465 nm and 665 nm as E4 and E6, respectively [27]. The ratio of E4/E6 and ΔlogK is often used to characterize the molecular weight of fulvic acid, which can reflect the complexity of its molecular structure, and it is inversely proportional to the molecular complexity and molecular weight: the higher the ratio of E4/E6 and ΔlogK, the simpler the molecular structure and the smaller the molecular weight. On the contrary, it indicates that the molecular structure is more complex [28,29,30]. The ratio of E4/E6 and the ΔlogK of the woody peat fulvic acid were the lowest at 0.230 and 1.643, respectively. The ratio of E4/E6 and the ΔlogK of herbaceous peat fulvic acid were the second highest at 0.275 and 1.750, respectively. Finally, the ratio of E4/E6 and the ΔlogK of mossy peat fulvic acid was the highest. The results revealed that the molecular weight of fulvic acid in woody peat was the highest, and its molecular structure was the most complex. Moreover, mossy peat had the lowest molecular weight of fulvic acid, with also the simplest structure.

### 2.3. Infrared and Visible Spectra of Different Types of Peat Fulvic Acids 

**Figure 3:**
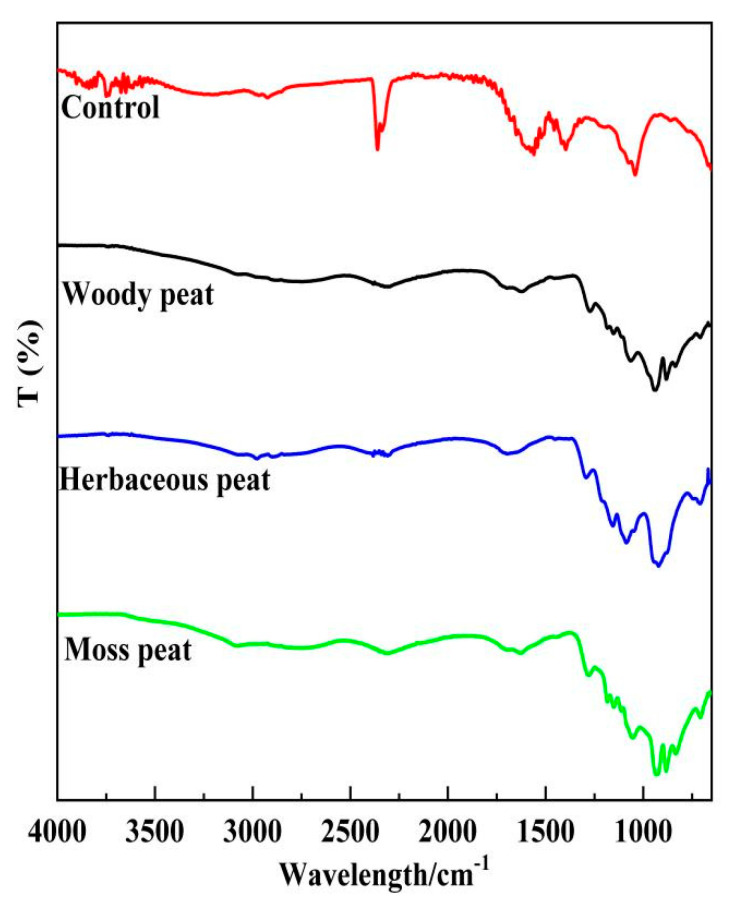
Infrared spectra of fulvic acid from herbaceous, woody, and mossy peats.

The infrared spectra of fulvic acid in herbaceous, woody, and mossy peats are shown in Figure 3. The infrared spectra revealed similar peak positions with differences in peak intensity (Table 2). The difference in the structures of fulvic acid in the different types of peat was primarily manifested in the content of functional groups, with little effect from the types of functional groups. The absorption peak near 3350 cm^−1^ was caused by a hydroxyl or amino stretching vibration, and the peak intensity trend was as follows: herbaceous peat fulvic acid > mossy peat fulvic acid > woody peat fulvic acid. These results indicate that herbaceous peat fulvic acid contains more hydroxyl and amino groups than the other peat fulvic acids. The stretching vibration absorption peaks of methyl and methylene were 2870 and 2850 cm^−1,^ respectively. The absorption peaks of mossy peat fulvic acid were the strongest, indicating that it contained more methyl and methylene groups than the other peat fulvic acids. The stretching vibration absorption peak of C=O in ketones, aldehydes, and carboxylic acids appeared near 1710 cm^−1^, and this peak of woody peat fulvic acid was the strongest, indicating that woody peat fulvic acid contains more carbonyl groups than other peat fulvic acids. The peak at 1560–1450 cm^−1^ is caused by the stretching vibration of the benzene ring. Woody peat fulvic acid had the strongest absorption peak at 1560–1450 cm−^1^, indicating that woody peat fulvic acid contained more benzene rings than the other two peat fulvic acids. The stretching vibration absorption peak of the ether bond was at 1033 cm^−1^, and this peak intensity from highest to lowest was in the order of herbaceous peat fulvic acid > woody peat fulvic acid > mossy peat fulvic acid, indicating that the ether bond content of herbaceous peat fulvic acid was higher than that of the other two peat fulvic acids. This is related to Aranganathan et al. [31], who are studying the data analysis of infrared spectroscopy of fulvic acid.

### 2.4. ^13^C Spectra of Different Types of Peat Fulvic Acids

**Figure 4:**
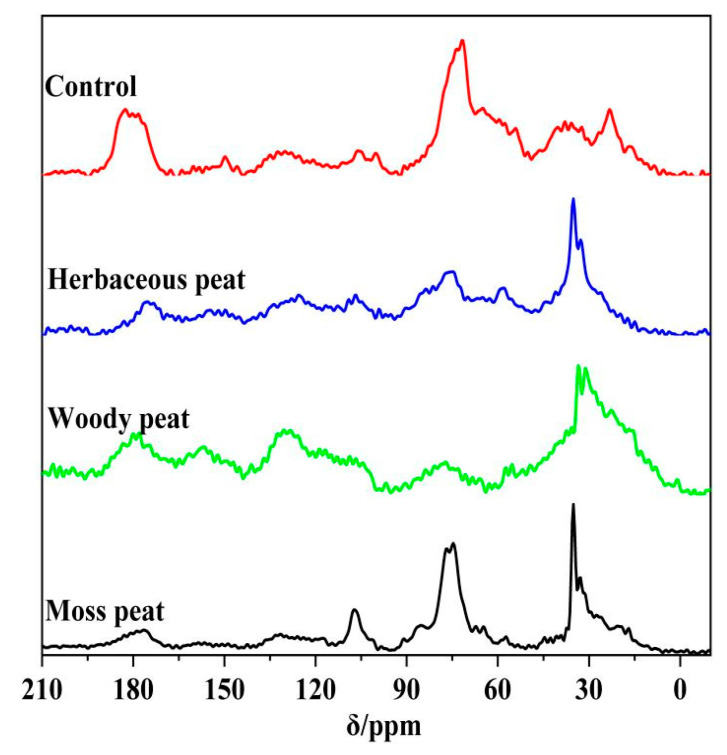
^13^C spectra of peat fulvic acid from herbaceous, woody and mossy plants.

Figure 4 shows the ^13^C-NMR spectra of the herbaceous, woody, and mossy peat fulvic acids. The spectra revealed that the displacement peaks of the different samples were approximately the same, and there were obvious displacement peaks in the ranges of 15–24, 32–24, 56–84, 120–-131, 140–160, and 170–180 ppm. The relative contents of various carbon-containing functional groups in the samples were obtained by integrating the ^13^C-NMR spectra of herbaceous, woody, and mossy peat fulvic acids, indicating that mossy peat fulvic acid contained more methyl and methylene than the other peat fulvic acids. The relative content of herbaceous peat fulvic acid was higher at 32–24 ppm, indicating that herbaceous peat fulvic acid contained more methylene than the other peat fulvic acids. The relative content of woody peat fulvic acid was higher in the ranges of 56–84, 120–131, and 140–160 ppm, indicating that woody peat fulvic acid contained more methoxy, aromatic carbon, aromatic ether, or phenolic substances than the other peat fulvic acids. In the 170–180 ppm region, only the herbaceous peat fulvic acid exhibited a peak, and woody and mossy peat fulvic acids had no peak in this region, indicating that only herbaceous peat fulvic acid contained carboxyl groups, and woody and mossy peat fulvic acid did not contain carboxyl groups, or the content was below the detection limit.

### 2.5. ^1^H Spectra of Different Types of Peat Fulvic Acid

**Figure 5:**
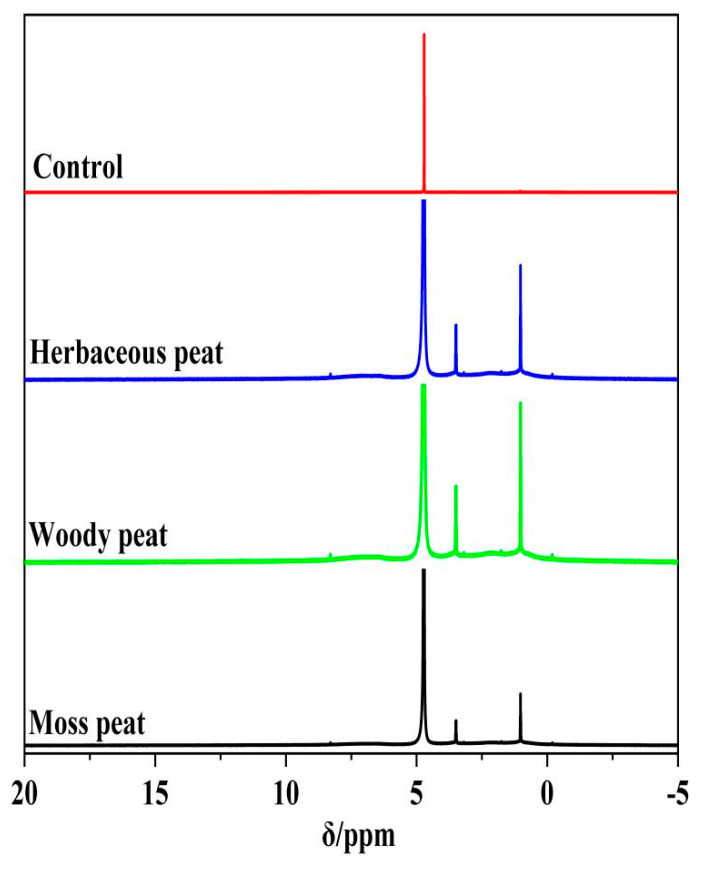
^1^H NMR spectra of fulvic acid in herbaceous, woody, and mossy peat.

Figure 5 shows the ^1^H NMR spectra of fulvic acid in herbaceous, woody, and mossy peats. According to literature reports, the attributions and relative contents of H in the ^1^H NMR spectroscopy results are shown in Table 3. The obtained H can be roughly classified into five categories: (1) 0.7–1.4 represents aliphatic saturated H, such as methyl methylene; (2) 3.5–4.0 signifies H on the methoxy group connected with oxygen; (3) 4.5–6.5 denotes H on C=C; (4) 6.4–7.2 represents H on the benzene ring; and (5) 8.3–9 represents phenolic hydroxyl H. According to Figure 5 and Table 4, the peak positions in the ^1^H NMR spectra of fulvic acid in herbaceous, woody, and mossy peats are roughly similar, with differences only in relative content. This result indicates that using different types of peat as raw materials had little effect on the type of fulvic acid H but affected the number of functional groups, which is similar to the results from the infrared analysis. In the ranges of 0.7–1.4 and 4.5–6.5 ppm, the relative content of mossy peat fulvic acid was high, indicating that mossy peat fulvic acid contained more methyl and methylene groups, as well as C=C, than the other peat fulvic acids. The relative content of woody peat fulvic acid was the highest at approximately 3.5–4.0 and 6.4–7.2 ppm, indicating that woody peat fulvic acid contained more methoxy group and benzene rings [32] than the other peat fulvic acids. The absorption of herbaceous peat fulvic acid was strongest within the range of 8.3–9.0 ppm, which is a characteristic shift of phenolic hydroxyl groups, indicating that herbaceous peat fulvic acid contained more phenolic hydroxyl groups than the other fulvic acid sources [33]. Wesley Machado et al. [10] also found the aromatic and aliphatic chemical structures of fulvic acid in nuclear magnetic resonance spectroscopy, but the structural differences are still very significant due to the different sources of fulvic acid.

### 2.6. XRD Spectra of Fulvic Acid in Different Types of Peat

**Figure 6:**
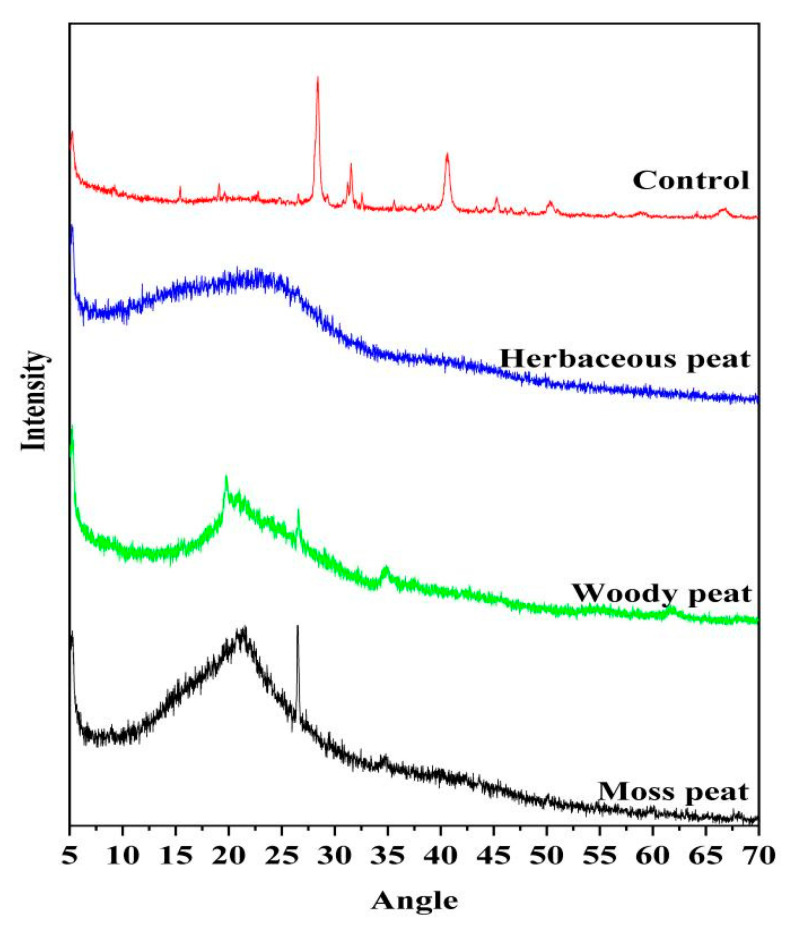
X-ray diffraction spectra of fulvic acid in herbaceous, woody, and mossy peats.

Figure 6 shows that the XRD spectra of herbaceous, woody, and mossy peat fulvic acids are similar. Compared with the XRD spectra of chromatographically pure fulvic acid, herbaceous, woody, and mossy fulvic acids were all serrated and disordered with rough lines, indicating that the extracted fulvic acid was amorphous and in low quantities. These results are consistent with the contents of extracted fulvic acid mentioned above, which was only 0.12–0.38%. Li Yanhong [34] reported that the crystalline and amorphous carbons of coal appear in the G (25°) and γ (21°) bands respectively. Moreover, herbaceous, woody, and mossy peat fulvic acids comprise aromatic and aliphatic carbons, and their molecular arrangements are disordered. Peat is formed by the physical or chemical processes of animal and plant remains, as well as other micro-organisms [35]. Therefore, chemical characterization may vary greatly depending on these factors. This is similar to the research results of Gong Guanqun et al. [36], who also found in the extraction experiment of humic acid that the functional groups of humic acid mainly contain carboxyl groups, hydroxyl groups, other oxygen-containing functional groups, and anhydride. It is possible that, due to different sources of humic acid, the types of functional groups obtained may still differ slightly.

### 2.7. XPS Spectra of Different Types of Peat Fulvic Acid

**Figure 7:**
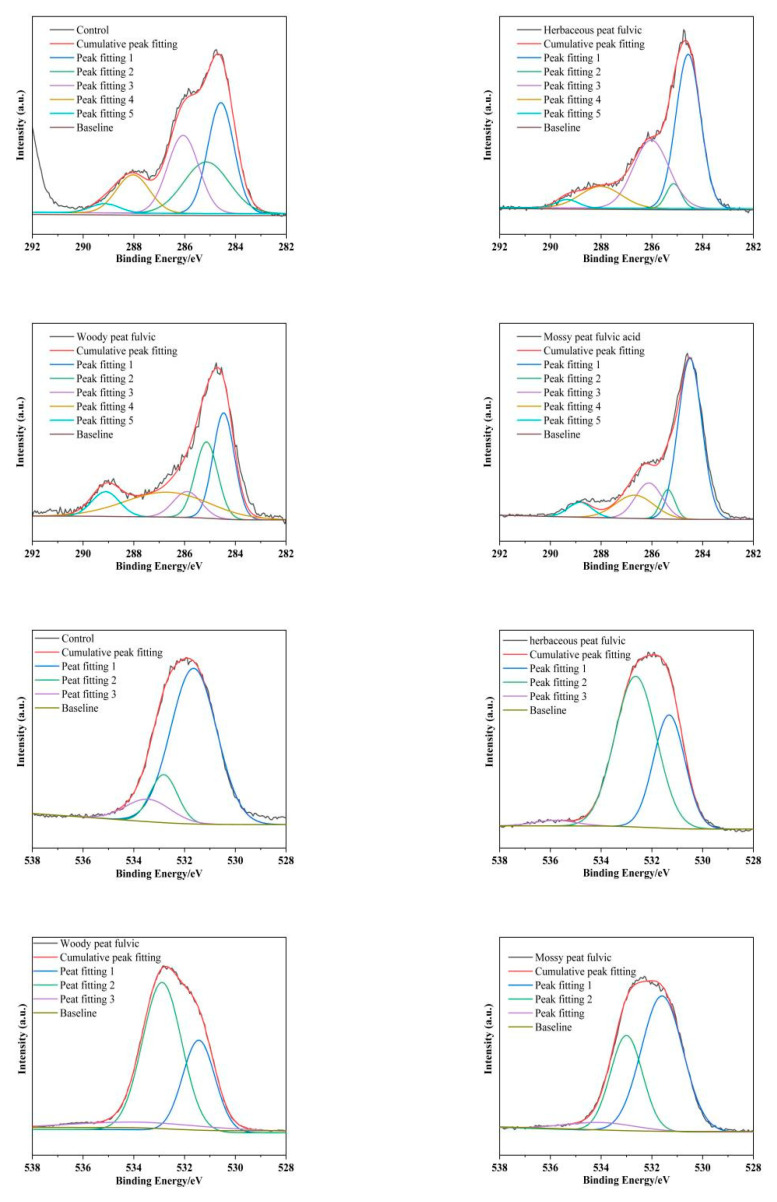
Peak-fitting C 1s and O 1s diagrams of fulvic acid in herbaceous, woody, and mossy peats.

The C and O elements in the C 1s peak-fitting diagrams of herbaceous, woody, and mossy peat fulvic acids are shown in Figure 7. The C 1s spectrum could be divided into five peaks: 284.8 ± 0.2, 285.1 ± 0.2, 285.8 ± 0.2, 287.5 ± 0.2, and 289.0 ± 0.2 eV, representing C–C, C–H, C–O, C=O, and –COOH, respectively. The content of C in the C–C form of fulvic acid in herbaceous, woody, and mossy peats showed little difference. Herbaceous peat fulvic acid contained more C=O and –COOH forms of C than the other peat fulvic acids. The O 1s spectrum of fulvic acid could be divided into three peaks: 531.5 ± 0.5, 532.5 ± 0.5, and 534.0 ± 0.5 eV, corresponding to C=O, C–O, and –COOH, respectively. Herbaceous, woody, and mossy peat fulvic acids did not contain any inorganic O. Of the three peat fulvic acids, woody peat fulvic acid contained more O elements in the form of C=O and C–O, and mossy peat fulvic acid had a higher O content in the form of –COOH. This is highly consistent with the XPS characterization of fulvic acid by Gong Guanqun et al [36]. Both studies believe that the main modes of carbon oxygen bonds in fulvic acid are C–O, –COOH, and C=O.

## 3. Materials and Methods

### 3.1. Materials

Herbaceous peat was purchased by Jilin Jixiang Peat Co. Ltd. (Jilin, China). Woody peat was provided by Hong Kong Zhongxiang International Ltd. (Hong Kong, China), and mossy peat was obtained from Qingdao Ouboya Horticultural Industry Co. Ltd. (Qingdao, China).

### 3.2. Experimental Methods

First, 10 g of 100-mesh herbaceous, woody, and mossy peats were placed into different beakers, and 40 mL of 5% NaOH was added (solid: liquid ratio of 1:4). After soaking for 24 h, 200 mL distilled water (solid: liquid ratio of 1:20) was added, and the reaction was heated and stirred at 80 °C for 2 h. Repeated centrifugal separation was used to obtain the supernatant. The pH of the filtrate was adjusted to 2–3 using a 5% H_2_SO_4_ solution, which was then layered after standing for 24 h. The supernatant (fulvic acid) was dried to a constant weight in an oven at 80 °C to obtain the desired product. Before drying, 1.00 mL of the supernatant was placed in a 100-mL conical flask, and 5.00 mL of 0.4 mol/L potassium dichromate and 15 mL of concentrated sulfuric acid were sequentially added. The solution was heated and oxidized in a boiling water bath for 30 min. After cooling to 25 °C, 3–5 drops of phenanthroline indicator solution were added. The solution was then titrated with ammonium ferrous sulfate. The color of the solution changed from orange to green to brick red, and the volume of ammonium ferrous sulfate consumed was recorded. Fulvic acid content (B; g/mL) and yield (A; %) were calculated as follows [37]:

$$B=\frac{0.003\times (V_{0}-V_{1})\times N}{C\times V}\times 100\%$$

$$A=\frac{B}{M}{\times V}_{2}\times 100\%$$

where 0.003 is the milligram equivalent of carbon (g), *V_0_* is the volume of ammonium iron(II) sulfate used (mL), *V_1_* is the volume of ammonium iron(II) sulfate consumed when titrating the sample (mL), *N* is the equivalent concentration of ammonium iron(II) sulfate (mol/L), *C* is the conversion coefficient of the carbon content ratio of fulvic acid (fulvic acid is 0.54), and *V* is the sample volume (mL). A is the extraction rate of fulvic acid, %; M is the mass of peat used for extracting fulvic acid, g.

### 3.3. Characterization Methods

#### 3.3.1. FTIR Spectroscopy

An FTIR spectrometer (TENSOR II, Bruker, Germany) was used to measure and record the light intensity of 1 mg of the dried fulvic acid sample. The determination conditions for all samples were consistent. The OMNIC (Version 9.2, Thermo Fisher Scientific, Waltham, MA, USA) software was used to analyze the data and calculate the relative size of the peak area.

#### 3.3.2. UV–Vis Light Analysis

The dried fulvic acid sample (10 mg) was accurately weighed, dissolved in water to 100 mL, and adjusted to pH 8.0 with 1% NaOH or 0.1 mol/L HCl to eliminate the influence of pH on the spectral measurement results. The UV–Vis spectrogram with a wavelength range of 200–800 nm was measured using a UV–Vis (CAYR 5000, Agilent, New York, NY, USA,) spectrophotometer with a resolution of 1 nm. The absorbance values at 465 and 665 nm were recorded and defined as E4 and E6, respectively, to obtain the ratio of E4/E6.

#### 3.3.3. XPS Analysis

Samples were analyzed using an ESCALAB250ZI X-ray photoelectron spectrometer (Schneider Electric Co., Ltd(China, Beijing)). The XPSPEAK(Version 9.2, Hong Kong, China) software was used to analyze the data and perform peak fitting.

#### 3.3.4. XRD Analysis

The D8Advance diffractometer (Bruker Co., Germany) was used for analysis, and the test conditions were as follows: target, Cu; voltage, 40 kV; current, 300 mA; diffraction angle, 2θ = 5–70°; and scanning speed, 5°/min [33].

#### 3.3.5. ^13^C-NMR Analysis

The ^13^C-NMR spectra of fulvic acid samples were obtained using an NMR spectrometer (Bruker AVANCE III 600M). The data were analyzed using the MestReNova software,(Version 14.0, mestrelab research, Santiago de Compostela, Spain), and the peak areas were integrated.

#### 3.3.6. ^1^H-NMR Analysis

The ^1^H-NMR spectra of fulvic acid, which was dissolved in deuterated H_2_O, were obtained using an NMR spectrometer (Bruker AVANCE III 600M). The data were analyzed using the MestRenova software(Version 14.0, mestrelab research, Santiago de Compostela, Spain), and the peak areas were integrated.

## 4. Conclusions

- Herbaceous peat had the highest fulvic acid yield, whereas the woody peat had the highest fulvic acid content. Therefore, herbaceous peat is the most suitable source for extracting fulvic acid.
- According to all the characterization methods in this experiment, the molecular differences in obtaining fulvic acid from the three different types of peat manifested in the content, not the types, of functional groups. In addition, the fulvic acid obtained from the three different types of peat comprised Aromatic and aliphatic carbon with a disordered molecular arrangement and no inorganic oxygen. Of the three peat fulvic acids, woody peat contained more oxygen in the forms of C=O and C–O, whereas mossy peat contained more oxygen in the form of carboxyl groups.
- Based on the various characterization methods mentioned above, the following conclusions can also be drawn. Herbaceous peat fulvic acid contained significant amounts of carbonyl, amino, methylene, carboxyl, and phenolic hydroxyl groups and ether bonds. Woody peat fulvic acid contains carbonyl and methoxy groups, benzene rings, aromatic carbons, aromatic ethers, and phenols. Mossy peat contained fulvic acid with the lowest molecular weight and simplest structure, and high quantities of methyl and methylene.
- This article studied the yield and content of humic acid extracted from peat, analyzed the molecular structure of three types of peat humic acid using different characterization methods, and reached the above conclusion. The research in this article has increased our understanding of humic acid extraction and can also provide reference for future scholars to study peat.

## Figures and Tables

**Table 1:** E4/E6 and ΔLogK values of fulvic acid in herbaceous, woody, and mossy peats.

	Herbaceous Peat	Woody Peat	Mossy Peat
E4	0.070	0.069	0.198
E6	0.040	0.042	0.062
E4/E6	1.750	1.643	3.129
ΔlogK	0.275	0.230	0.545

**Table 2:** Comparison of the infrared peak positions.

Peak Position	Functional Groups	Absorption Type
3350 cm^−1^	–OH	extensional vibration
2870 cm^−1^	–CH_3_	stretch vibration
2850 cm^−1^	–CH_2_-	stretch vibration
1710 cm^−^1	–C=O	extensional vibration
1560–1450 cm^−1^	C–C bond on benzene	extensional vibration
1033 cm^−1^	C–O on alcohols, phenols and ethers	extensional vibration

**Table 3:** Attributions and relative contents of functional groups with different displacements of fulvic acid in herbaceous, woody, and mossy peats (CNMR).

	15–24 Methyl Carbon	32–42 Methylene Carbon	65–90 Methoxy Group Carbon	90–140 Aromatic Charcoal	140–160 Aromatic C–O	160–190 Carboxyl Carbon	Aromaticity Index
Herbaceous peat fulvic acid	1.19	0.78	12.10	0.99	0.15	0.41	1.14
Woody peat fulvic acid	0.09	1.00	33.00	1.24	0.89	/	2.13
Mossy peat fulvic acid	1.68	11.98	3.46	0.17	0.63	/	0.80

**Table 4:** Attributions and relative contents of functional groups with different displacements of fulvic acid in herbaceous, woody, and mossy peats (HNMR).

	0.7–1.4 Methyl Methylene Hydrogen	3.5–40 Methoxy Group Hydrogen	4.5–6.5 Carbon–Carbon Double Bond Hydrogen	6.4–7.2 Benzene Ring Hydrogen	8.3–9 Phenolic Hydroxyl Hydrogen
Herbaceous peat fulvic acid	1.06	0.91	8.03	0.09	0.15
Woody peat fulvic acid	1.14	1.26	21.06	4.20	0.08
Mossy peat fulvic acid	5.56	1.01	23.99	2.42	0.04

## Data Availability

Samples of the compounds are available from the authors.
